# Complete Genome Sequence of *Neisseria weaveri* Strain NCTC13585

**DOI:** 10.1128/genomeA.00815-16

**Published:** 2016-08-25

**Authors:** Sarah Alexander, Mohammed-Abbas Fazal, Edward Burnett, Ana Deheer-Graham, Karen Oliver, Nancy Holroyd, Julian Parkhill, Julie E. Russell

**Affiliations:** a Culture Collections, Public Health England, London, United Kingdom; b Wellcome Trust Sanger Institute, Wellcome Trust Genome Campus, Hinxton, United Kingdom

## Abstract

*Neisseria weaveri* is a commensal organism of the canine oral cavity and an occasional opportunistic human pathogen which is associated with dog bite wounds. Here we report the first complete genomic sequence of the *N. weaveri* NCTC13585 (CCUG30381) strain, which was originally isolated from a patient with a canine bite wound.

## GENOME ANNOUNCEMENT

*Neisseria weaveri* is a Gram-negative, nonmotile, aerobic, rod-like bacterium that is a commensal of the canine oral flora (1–3). It is an infrequent opportunistic pathogen of dog bite wounds and there are also isolated reports of *N. weaveri* causing lower respiratory tract and septic infection (4, 5). There is a paucity of information on *N. weaveri* within the literature and historically there has been confusion with its taxonomy, with two different type strains being described (1–3). To date there are only two draft genomes available for *N. weaveri*, LMG 5135 and ATCC 51223 (both the proposed type strains), which have been assembled into 46 and 40 contigs, respectively (6, 7). Here we report the first complete genome for *N. weaveri* strain NCTC13585, which was isolated from a human dog bite wound in Danderyd, Sweden, in 1982.

High-molecular-weight genomic DNA was extracted from a pure culture using the Masterpure DNA extraction kit (Epicentre, WI, USA) and the quality was confirmed (>50 kb) using the Agilent 2200 TapeStation. Whole-genome sequencing (WGS) was performed using the PacBio single-molecule real-time (SMRT) DNA sequencing technology utilizing C4/P6 chemistry followed by genome assembly using an automated assembly pipeline and annotation with Prokka.

The genomic size of NCTC13585 was determined to be 2,188,497 bp. The average G+C content of the sequence was 49.0%. No plasmids were identified in this strain. In total there were 2,060 coding sequences, four rRNA operons, and 55 tRNA genes identified.

### Accession number(s).

The complete genome sequence has been deposited in the European Nucleotide Archive under the BioSample accession number SAMEA3174300 and the assembly accession number LT571436.
